# Repeat polymorphisms in the *Homo sapiens* heme oxygenase-1 gene in diabetic and idiopathic gastroparesis

**DOI:** 10.1371/journal.pone.0187772

**Published:** 2017-11-21

**Authors:** Simon J. Gibbons, Madhusudan Grover, Kyoung Moo Choi, Akhilesh Wadhwa, Adeel Zubair, Laura A. Wilson, Yanhong Wu, Thomas L. Abell, William L. Hasler, Kenneth L. Koch, Richard W. McCallum, Linda A. B. Nguyen, Henry P. Parkman, Irene Sarosiek, William J. Snape, James Tonascia, Frank A. Hamilton, Pankaj J. Pasricha, Gianrico Farrugia

**Affiliations:** 1 Mayo Clinic, Enteric NeuroScience Program, Rochester, Minnesota, United States of America; 2 Johns Hopkins University Bloomberg School of Public Health, Johns Hopkins University, Baltimore, Maryland, United States of America; 3 Mayo Clinic, Medical Genomics Program, Rochester, Minnesota, United States of America; 4 University of Louisville, Louisville, Kentucky, United States of America; 5 University of Michigan, Ann Arbor, Michigan, United States of America; 6 Wake Forest University, Winston-Salem, North Carolina, United States of America; 7 Texas Tech University, El Paso, Texas, United States of America; 8 Stanford University, Palo Alto, California, United States of America; 9 Temple University, Philadelphia, Pennsylvania, United States of America; 10 California Pacific Medical Center, San Francisco, California, United States of America; 11 National Institute of Diabetes and Digestive and Kidney Diseases, Bethesda, Maryland, United States of America; 12 Johns Hopkins School of Medicine, Baltimore, Maryland, United States of America; University of Missouri Health Care, UNITED STATES

## Abstract

**Background:**

Idiopathic and diabetic gastroparesis in *Homo sapiens* cause significant morbidity. Etiology or risk factors have not been clearly identified. Failure to sustain elevated heme oxygenase-1 (HO1) expression is associated with delayed gastric emptying in diabetic mice and polymorphisms in the HO1 gene (*HMOX1*, NCBI Gene ID:3162) are associated with worse outcomes in other diseases.

**Aim:**

Our hypothesis was that longer polyGT alleles are more common in the *HMOX1* genes of individuals with gastroparesis than in controls without upper gastrointestinal motility disorders.

**Methods:**

Repeat length was determined in genomic DNA. Controls with diabetes (84 type 1, 84 type 2) and without diabetes (n = 170) were compared to diabetic gastroparetics (99 type 1, 72 type 2) and idiopathic gastroparetics (n = 234). Correlations of repeat lengths with clinical symptom sub-scores on the gastroparesis cardinal symptom index (GCSI) were done. Statistical analyses of short (<29), medium and long (>32) repeat alleles and differences in allele length were used to test for associations with gastroparesis.

**Results:**

The distribution of allele lengths was different between groups (P = 0.016). Allele lengths were longest in type 2 diabetics with gastroparesis (29.18±0.35, mean ± SEM) and longer in gastroparetics compared to non-diabetic controls (28.50±0.14 vs 27.64±0.20 GT repeats/allele, P = 0.0008). Type 2 diabetic controls had longer alleles than non-diabetic controls. In all gastroparetic groups, allele lengths were longer in African Americans compared to other racial groups, differences in the proportion of African Americans in the groups accounted for the differences between gastroparetics and controls. Diabetic gastroparetics with 1 or 2 long alleles had worse GCSI nausea sub-scores (3.30±0.23) as compared to those with 0 long alleles (2.66±0.12), P = 0.022.

**Conclusions:**

Longer poly-GT repeats in the *HMOX1* gene are more common in African Americans with gastroparesis. Nausea symptoms are worse in subjects with longer alleles.

## Introduction

Gastroparesis causes symptoms of post-prandial fullness, early satiety, nausea, vomiting and bloating [1] and also abdominal pain in many patients [2]. It is a disabling disorder causing high levels of morbidity and increased mortality in affected people [3]. Gastroparesis is a well-recognized complication of diabetes [4, 5], more commonly seen in patients with type 1 than type 2 diabetes [6] and appears to associate with acute and chronic hyperglycemia in patients with type 1 diabetes [7]. Other forms of gastroparesis are associated with certain medications, including opiates and glucagon-like peptide-1 (GLP-1) analogs, and as a complication of some gastric surgeries but a large proportion of patients have idiopathic gastroparesis [8].

It is unclear why gastroparesis, like many other diabetic complications, develops in only a subset of diabetics [9]. Genetic susceptibility plays a role in diabetic retinopathy, nephropathy and coronary artery calcification [10–12]. However, there are no published studies investigating familial associations in gastroparesis. Similarly, long-term epigenetic modification of the chromatin has also been linked to microvascular complications of diabetes including retinopathy and nephropathy [13] but has not been investigated for gastroparesis.

For this study, we investigated heme oxygenase 1 (HO1) as a possible susceptibility factor for gastroparesis due to evidence from animal models of diabetic gastroparesis showing that elevated HO1 expression protects diabetic mice from development of delayed gastric emptying [14]. Additionally, a long repeat polymorphism in the promoter of the gene encoding HO1, *HMOX1* (NCBI Gene ID:3162) associates with worse outcomes in several diseases [15–18] including coronary artery disease in people with type 2 diabetes [19]. The polymorphism is a dinucleotide (GT)n repeat that is between 200 and 250 nucleotides upstream of the first exon of *HMOX1*, which contains between 15 and 40 GT repeats [20]. This sequence is typical of other dinucleotide repeat sequences that are variable in length, are more common in regions of active gene transcription and are associated with enhancer regions in genes [21, 22]. Long poly dinucleotide repeats have the capacity to assume the Z-DNA conformation with a left handed double helix structure and the ability to inhibit gene transcription [23]. For the *HMOX1* promoter, sequences containing longer polyGT repeats had lower transcriptional activity than sequences with fewer repeats [16, 19], meaning that shorter polyGT repeat alleles result in higher expression of HO1 protein [24]. Previous studies examining HO1 expression in the gastric muscularis propria of diabetic mice found low levels of HO1 in mice with delayed gastric emptying and high levels of HO1 in diabetic mice with normal gastric emptying [14]. Induction of HO1 expression by treatment with hemin or interleukin-10 (IL-10) of diabetic mice with delayed gastric emptying normalized gastric emptying [14, 25]. Therefore the *a priori* hypothesis of this study was that longer polyGT alleles are more common in the *HMOX1* genes of individuals with gastroparesis than in control subjects without upper gastrointestinal motility disorders. We also set out to examine whether any specific symptoms of gastroparesis are associated with the longer polyGT repeat allele in the promoter of the *HMOX1* gene.

To test this hypothesis the specific aim was to sequence the promoter region of genomic DNA from blood of individuals without diabetes and with no symptoms of gastroparesis, individuals with diabetes and no symptoms of gastroparesis, individuals with diabetic gastroparesis and individuals with idiopathic gastroparesis. Possible associations were investigated between polyGT allele length in *HMOX1* genes and type of diabetes, levels of HbA1c and specific symptoms of gastroparesis defined by the gastroparesis cardinal symptom index (GCSI) [26]. Differences in polyGT allele length were examined between black/African American individuals and non-black individuals in the gastroparetic groups since there is evidence that African Americans with gastroparesis are reported to have more severe symptoms that members of other racial groups [27].

## Materials and methods

Gastroparesis subjects were enrolled by the 8 clinical sites of the National Institutes of Health’s Gastroparesis Clinical Research Consortium (GpCRC). Enrollment was covered by local Institutional Review Boards. Genomic DNA, isolated from whole blood, was obtained from the GpCRC subjects and from controls from the Mayo Clinic Biobank and Mayo Clinic Center for Cell Signaling in Gastroenterology Clinical Core. All participants provided written consent. The study was approved by the Mayo Clinic Institutional Review Board and was registered (ClinicalTrials.gov NCT00398801).

### Subject enrollment: Patients with gastroparesis

The gastroparesis subjects were adults (> 18 years old) with delayed gastric emptying on scintigraphy (>60% retention at 2 hours or > 10% retention at 4 hours) and with no evidence of gastric outlet obstruction for at least 12 weeks. Presence of active inflammatory bowel disease, eosinophilic gastroenteritis, neurological conditions, acute liver or renal failure, or a history of total or subtotal gastric resection was used as exclusion criteria. At enrollment, participants completed validated symptom questionnaires, upper endoscopy, gastric emptying and laboratory tests. Symptoms of gastroparesis were assessed from the results of a 20-item Patient Assessment of Upper Gastrointestinal Disorders Symptoms Severity Index (PAGI-SYM) questionnaire and we used the data on the symptoms that form the Gastroparesis Cardinal Symptom Index [26] for this study, specifically the overall GCSI, the nausea/vomiting sub-score, postprandial fullness/early satiety sub-score and bloating sub-score. These symptoms were scored on a 0–5 Likert scale. For more details see Parkman et al. [28]. Gastric emptying was measured by scintigraphy and glycemic control was assessed by glycosylated hemoglobin levels (HbA1c) in the blood as reported previously [29].

### Subject enrollment: Controls

DNA samples from control subjects with diabetes but no symptoms of gastroparesis were obtained from the Mayo Clinic Biobank. This is a repository of biological specimens and connected medical records from adult Mayo Clinic patients who are US resident and who consent to provide a blood sample as well as access to other tissue samples and their electronic medical record [30]. Genomic DNA was obtained from patients with a history of either type 1 or type 2 diabetes and no history of gastroparesis or related upper gastrointestinal disorders (functional dyspepsia, unexplained nausea and vomiting). Duration of the diabetes at the time of blood collection was obtained where feasible. The control group was sex matched to the proportions in the diabetic gastroparesis group. Samples from control subjects without diabetes and without symptoms of gastroparesis were obtained from the Mayo Clinic Center for Signaling in Gastroenterology Clinical Core. These samples were collected from adults receiving colonoscopic examinations at the Mayo Clinic Rochester between 1995-to the present and de-identified clinical information was abstracted.

The controls were chosen to be older than the gastroparetic subjects and, for diabetics, to have a sufficient duration of diabetes to give opportunity for the genetic variation to be expressed as a phenotypic difference. As a result, all groups of controls were significantly older than the respective gastroparesis subjects. Race/ethnicity was self-reported by the subjects in this study.

### DNA sequencing

The length of the polyGT alleles in the *HMOX1* genes for each subject was determined by PCR amplification and capillary electrophoresis from genomic DNA on the ABI3730 platform (Applied Biosystems^™^, Foster City, CA) using the following primers: 5’-AGAGCCTGCAGCTTCTCAGA-3’ and 5’-ACAAAGTCTGGCCATAGGAC-3’. Allele length was defined as short (≤28 GT repeats), medium (29–32 repeats) and long (≥ 33 repeats).

### Statistical analysis and power calculations

Sample sizes were chosen on the basis of reported proportions of patients with short, medium or long poly GT allele lengths revealing that 5% of control patients have long length alleles [19, 31]. We calculated that 82 subjects per group will allow a 15% effect size determination for differences in long allele length prevalence between the gastroparesis and control groups with 80% power based on a two-sided test significance of 0.05. Distributions of continuous variables in groups are reported as means ± SEM and compared by parametric statistical tests, either Student’s t tests or one way analysis of variance (ANOVA) with Tukey’s post-tests for multiple comparisons. Categorical variables are reported as medians with inter-quartile ranges and were compared by non-parametric statistical tests, either Mann Whitney or Kruskal-Wallis ANOVA with Dunn’s post-tests. Differences in proportions were analyzed by Chi-squared tests. Associations between allele length and other variables was determined by plotting the sum of the two allele lengths in each subject against each of the measured variables and then fitting the data by linear regression using a least squares algorithm, if the slope of the fit was significantly different from zero based on the F test then an association was considered to be present. Significance was taken as P < 0.05 and calculated in Graphpad Prism (GraphPad software Inc., La Jolla, California).

## Results

### Demographic characteristics and clinical data

Tables 1 and 2 provide demographic characteristics of the four groups. There were more females (82.5%) in the gastroparesis groups, especially in the idiopathic gastroparesis group consistent with the reported gender distribution for this disease [32]. The non-diabetic control group contained a significantly lower proportion of females (52.4%). The diabetic control groups had a similar proportion of females (70.2%) compared to the diabetic gastroparesis groups (73.1%). The controls (non-diabetic, non-gastroparetic and diabetic, non gastroparetic) were significantly older than the respective cases (idiopathic gastroparesis and diabetic gastroparesis). Both type 1 and type 2 diabetic controls were older than the corresponding gastroparesis subjects (all P<0.001, one-way ANOVA with Tukey’s Post-test). The idiopathic gastroparetic subjects were significantly younger than the diabetic gastroparetic subjects (P<0.001, one-way ANOVA with Tukey’s Post-test) but there were no age differences between those and the sub-group of type 1 diabetes gastroparetics. The subjects in the control groups were predominantly white, non-Hispanics, whereas a significant proportion of the subjects in the gastroparetic groups identified as Black and/or African American. The proportion of non-whites was significantly higher in the diabetic gastroparesis group (21.1%) than in the idiopathic gastroparesis group (3.0%, P<0.0001, Chi^2^ test) and compared to the control groups (1.2%, P<0.0001, Chi^2^ test).

**Table 1:** Data on subjects that were studied.

	Non diabetic, Non Gastroparetic Controls (n = 170)	Diabetic, Non Gastroparetic Controls (n = 168)	Diabetic Gastroparetic (n = 171)	Idiopathic Gastroparetic (n = 234)
**Sex (Females)**	89	118	125	209
**Age (median, IQR)**	72, 64–78 *	64, 53–73 §	45, 36–55.5 *	39, 29–51 #
**Diabetes**				
**Type 1**		84	99	
**Type 2**		84	72	
**Diabetes duration (Yrs, median, IQR)**	-	13.45, 9.03–15.34	-	-
**BMI (kg/cm**^**2**^**)**	-	29.52, 25.82–34.44	-	-

Averages are shown as medians with the inter-quartile range. Statistical differences were determined by one-way ANOVA with Tukey’s post-test or by a Kruskal-Wallis test with a Dunn’s post-test and are indicated as P < 0.05 for * vs idiopathic gastroparesis, § vs diabetic gastroparetic, # vs type II diabetic controls.

**Table 2:** Data on gastroparetic subjects that were studied.

	Type 1 Diabetic Gastroparetic	Type 2 Diabetic Gastroparetic	Idiopathic Gastroparetic
**Numbers**	99	72	234
**Male**	31	15	25
**Female**	68	57	209
**Ethnicity: Hispanic**	15	10	7
**Race: White**	75	60	227
** Black**	20	10	15
** Asian**	1	1	1
** Indian/Native American**	3	2	4
**Age**	39, 29.5–48 $	53.5, 45–59.25 #	39, 29–51
**Gastric retention 2 hr**	72, 54–84 *	60.95, 42.2–76.325 $	64, 51–75
**Gastric retention 4 hr**	36, 21.4–62 *	24.65, 16–41.975 $	23, 14.3–39.9 §
**Ave Nausea Sub-score**	3, 1.33–4	2.67, 1–3.67	2.33, 1.33–3.67
**Ave Fullness Sub-score**	3.25, 2.375–4 *	3.25, 2.6875–4	3.75, 3–4.25 §
**Ave Bloating Sub-score**	3, 1.5–4	3.5, 2–4.5	3, 2–4
**Ave GCSI**	3.03, 2.17–3.625	3.139, 2.2083–3.9306	3.069, 2.361–3.67
**HbA1c**	8.2, 7–9.45 $*	7.1, 6.1–8.4 *	5.4, 5.2–5.6 §

Averages are shown as medians with the inter-quartile range. Statistical differences were determined by one-way ANOVA with Tukey’s post-test or by a Kruskal-Wallis test with a Dunn’s post-test and are indicated as P < 0.05 for * vs idiopathic gastroparesis, § vs diabetic gastroparetic, $ vs type I diabetic controls, # vs type II diabetic controls.

At the time of blood collection, the type 2 diabetic controls had been diabetic for significantly longer (13.85, 11.54–15.76 yrs, Median, IQR) duration than the type 1 diabetic controls (11.48, 4.47–15 yrs, median, IQR, P<0.0004, Mann Whitney test). As expected, HbA1c levels were elevated in patients with diabetes and significantly different between the groups (P<0.0001, Kruskal Wallis test, Table 2). Subjects with idiopathic gastroparesis had HbA1c levels in the normal range (4.6–6.3%) whereas all groups of subjects with diabetes and gastroparesis had significantly higher levels than those with idiopathic gastroparesis and subjects with type 1 diabetes and gastroparesis had higher HbA1c levels than subjects with type 2 diabetes and gastroparesis (Table 2).

The overall GCSI was not significantly different between the gastroparesis groups. For the sub-scores, with respect to fullness, there was a difference between the groups (P = 0.0048, Kruskal Wallis test, Table 2); subjects with idiopathic gastroparesis had significantly worse symptoms of fullness than patients with diabetic gastroparesis and the difference was also significant between subjects with idiopathic gastroparesis and those with type 1 diabetes and gastroparesis (Table 2).

There were significant differences in gastric emptying between the groups at two hours and at four hours (Table 2). At both time points, type 1 diabetes gastroparesis subjects had significantly less gastric emptying than type 2 diabetic gastroparesis subjects. Subjects with idiopathic gastroparesis had significantly more gastric emptying than subjects with type 1 diabetes and gastroparesis (P<0.0001, Kruskal Wallis test, Table 2). At four hours, gastric emptying values from all subjects with diabetic gastroparesis were significantly lower than the values in subjects with idiopathic gastroparesis (Table 2).

### Distribution of HMOX1 polyGT allele lengths and genotypes

**Fig 1:**
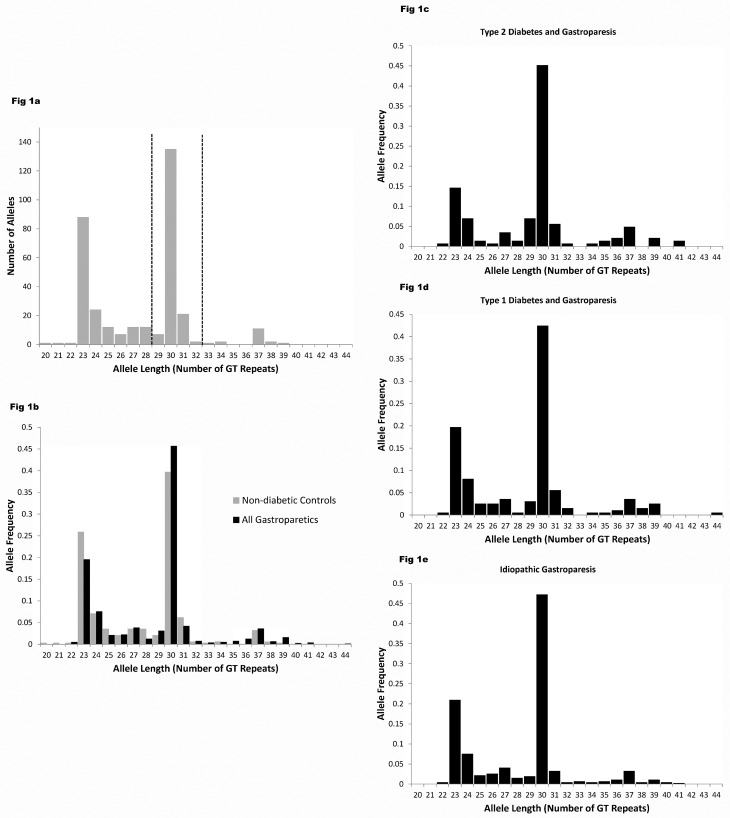
Frequency distribution of the lengths of the polyGT repeat allele in the *HMOX1* gene. a) Frequency distribution of the lengths of the polyGT repeat allele in the *HMOX1* gene from control subjects with no diabetes and no symptoms of gastroparesis. Dotted lines indicate division chosen between short, medium and long alleles. b) Fractional frequency distribution of the lengths of polyGT repeat in *HMOX1* from control, non-diabetic subjects (n = 170) compared to all gastroparetic subjects (n = 576). The distribution of the allele lengths was significantly different between the groups (P = 0.004, Mann Whitney test). c) Distribution of allele lengths in subjects with type 2 diabetes and gastroparesis, d) type 1 diabetes and gastroparesis, e) idiopathic gastroparesis. Allele length was determined by PCR amplification of genomic DNA from blood using the ABI 3730 platform and capillary electrophoresis and using primers flanking the GT repeat region.

The distribution of the polyGT allele lengths in the *HMOX1* gene from non-diabetic controls extended from 20 to 39 repeats and appeared to fall into 3 clear groups (Fig 1a) as previously reported by others [33–35]. For the non-diabetic, non-gastroparetic controls the distribution of these alleles was not significantly different from Hardy Weinberg equilibrium (Chi^2^ P = 0.32). For gastroparetic subjects, 3 groups of allele lengths were also observed (Fig 1b) but there was a significant shift in the distribution of the allele lengths towards longer lengths in gastroparetic subjects compared non-diabetic controls (P = 0.004, Mann Whitney test). As a result, the mean allele length (GT repeats per allele) was longer in gastroparetic subjects (28.50 ± 0.14, n = 405) than non-diabetic controls (27.64 ± 0.20, n = 170), (P = 0.0008, t test).

The distribution of polyGT allele lengths was also significantly different between subsets of gastroparetic subjects (P = 0.016, Kruskal Wallis ANOVA) (Fig 1c, 1d and 1e). Allele lengths were longest in subjects with type 2 diabetes and gastroparesis (Fig 1c, 29.18 ± 0.35 GT repeats per allele, n = 72, P < 0.05 Dunn’s post-test vs non-diabetic controls) but were not significantly longer when comparing controls to subjects with type 1 diabetes and gastroparesis (Fig 1d, 28.58 ± 0.31 GT repeats per allele, n = 99) or to subjects with idiopathic gastroparesis (Fig 1e, 28.26 ± 0.18 GT repeats per allele, n = 234).

**Fig 2:**
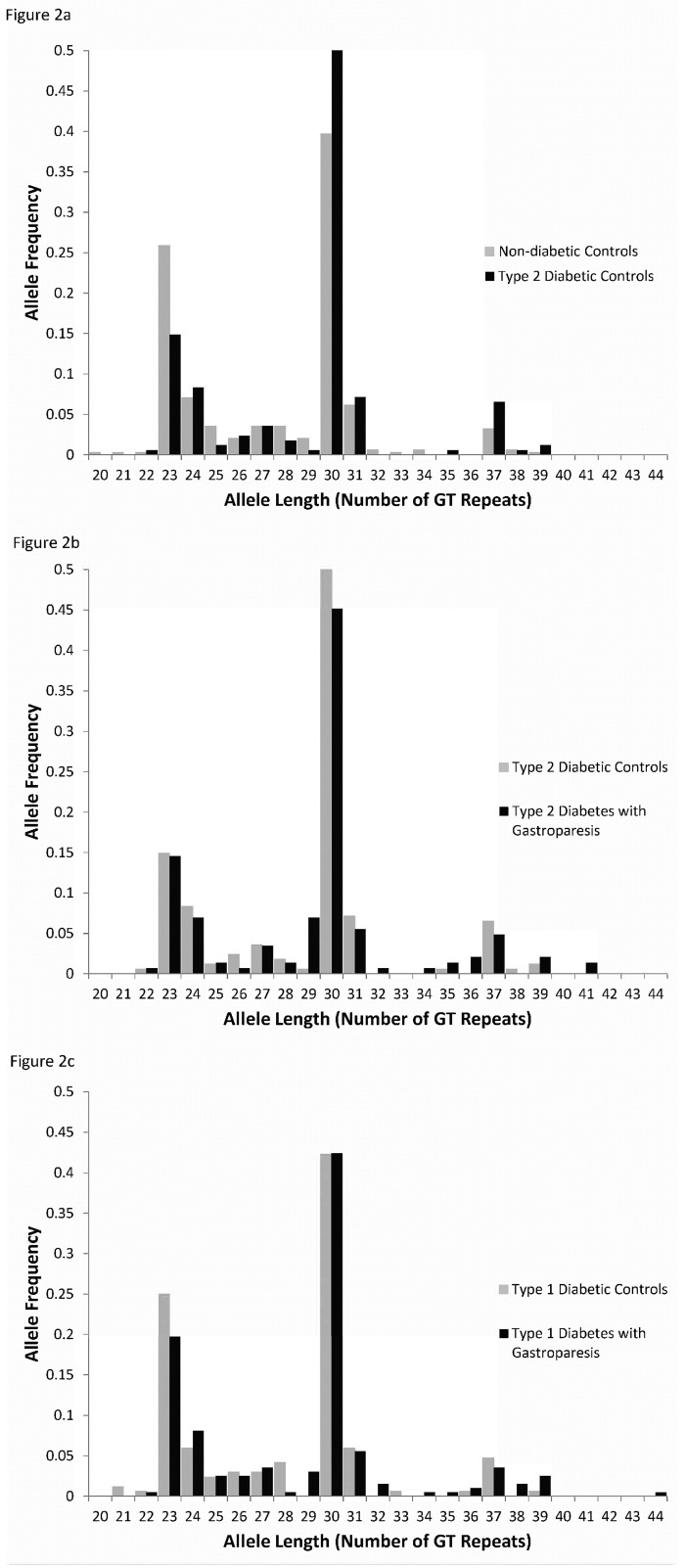
Size distribution of the polyGT repeat lengths. a) The size distribution of the polyGT repeat length in type 2 diabetic controls is significantly longer than the allele length in non-diabetic controls. The fractional frequency distribution of the lengths of polyGT repeat in *HMOX1* from non-diabetic control subjects with no GI motility disorders (n = 170) compared to subjects with type 2 diabetes and no GI motility disorders (n = 84) are shown (P < 0.05 Dunn’s test). b) Fractional frequency distribution of the lengths of polyGT repeat in *HMOX1* from subjects with type 2 diabetes and no GI motility disorders (n = 84) compared to subjects with type 2 diabetes and gastroparesis (n = 72, P = NS, Dunn’s test). c) Fractional frequency distribution of the lengths of polyGT repeat in *HMOX1* from subjects with type 1 diabetes and no GI motility disorders (n = 84) compared to subjects with type 1 diabetes and gastroparesis (n = 99, P = NS, Dunn’s test). Allele length was determined by PCR amplification of genomic DNA from blood using the ABI 3730 platform and capillary electrophoresis and using primers flanking the GT repeat region.

To investigate whether this is an association with type 2 diabetes or with gastroparesis, we studied two groups of diabetics but no reported symptoms of gastroparesis or related disorders (functional dyspepsia or chronic nausea and vomiting). We found no significant differences between the groups of type 2 diabetics with or without gastroparesis (P > 0.05, Dunn’s test, Fig 2b). Additionally, polyGT repeat lengths were not significantly different between patients with type 1 diabetes but no gastric disorders and non-diabetic controls or subjects with type 1 diabetes and gastroparesis (P > 0.05, Dunn’s test, Fig 2c). However, six of our gastroparesis subjects had very long alleles (> 39 polyGT repeats) which was not seen in any of the controls regardless of their diabetic status (see Fig 1b).

**Fig 3:**
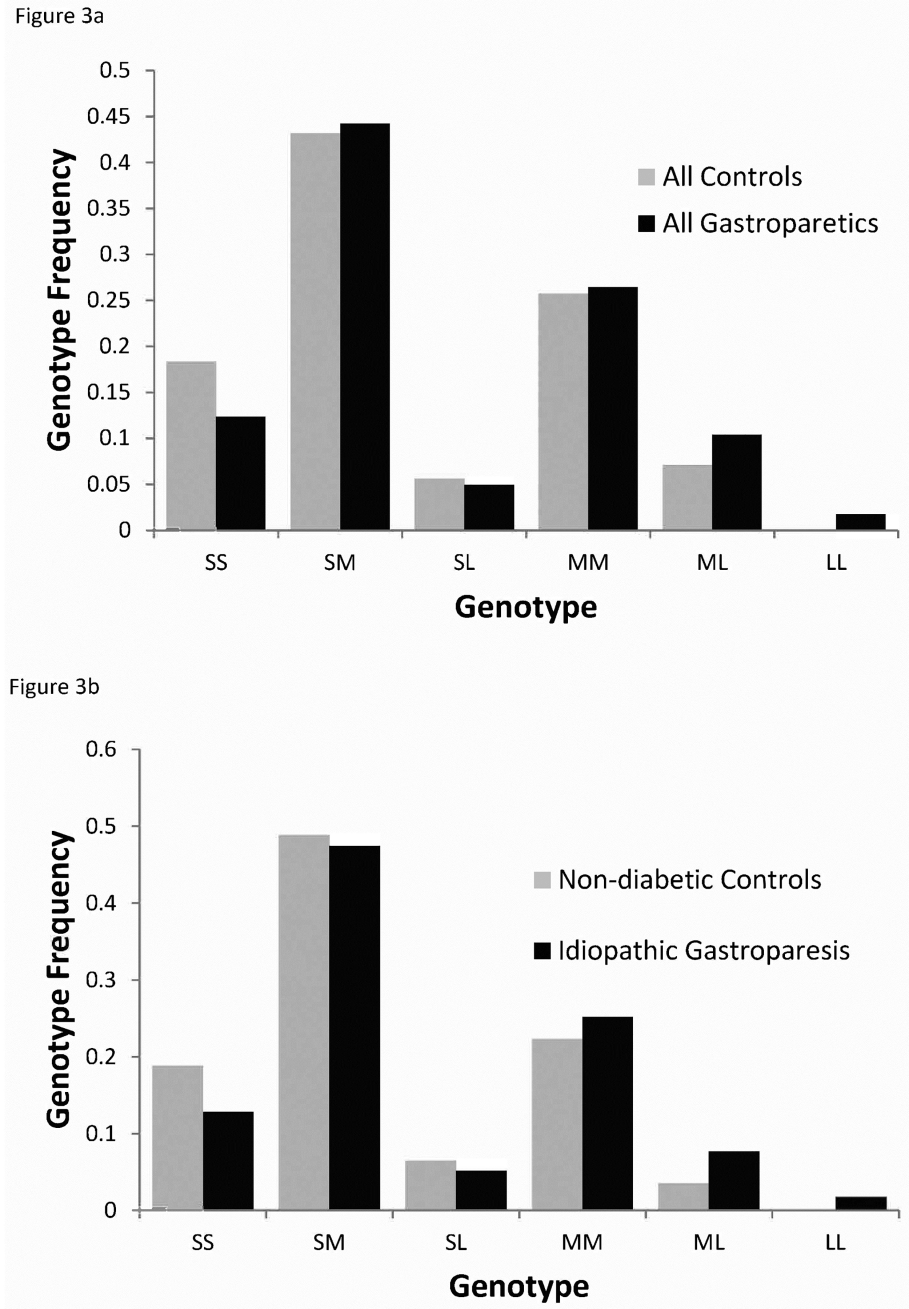
Allele distribution in gastroparetic and control subjects. Allele distribution of long (L), medium (M) and short (S) alleles a) for subjects with gastroparesis is significantly different from the distribution for control subjects (Chi^2^ P value = 0.019) and b) for subjects with idiopathic gastroparesis compared to non-diabetic controls (Chi^2^ P value = 0.049). Short alleles were defined as shorter than 29 polyGT repeats and long alleles were defined as longer than 32 repeats (see Fig 1a for definition).

For the genotypes of the subjects, no control subjects were homozygous for the long allele but 7 gastroparetic subjects were LL for the polyGT repeat and as a result, the distribution of the genotypes was significantly different not only between all control subjects compared to all subjects with gastroparesis (Chi^2^ P value = 0.019, Fig 3a) and between all controls compared to type 2 diabetics with gastroparesis (Chi^2^ P value = 0.0071) but also between non-diabetic control subjects compared to subjects with idiopathic gastroparesis (Chi^2^ P value = 0.049, Fig 3b). In addition, we found that the distributions of the short, medium and long alleles were not in Hardy Weinberg equilibrium for subjects with idiopathic gastroparesis (Chi^2^ P value = 0.04).

### Racial disparities in the distribution of the HMOX1 polyGT allele lengths in individuals with gastroparesis

It is reported that African Americans with HIV have longer polyGT repeat alleles in the *HMOX1* gene than Caucasians with HIV [36]. We compared the average allele length for individuals who identified as black and/or African American compared to the other racial groups in the groups of subjects with gastroparesis. The polyGT repeat alleles were significantly longer in the HMOX1 genes of black/African American individuals compared to other racial groups for all gastroparetics (Black: 31.52 ± 0.55, Non-black: 28.13 ± 0.14, P = 0.0001, Kruskal-Wallis with Dunn’s post-test), idiopathic gastroparetics (Black: 31.83 ± 0.95, Non-black: 28.02 ± 0.18, P = 0.005, Kruskal-Wallis with Dunn’s post-test) and diabetic gastroparetics (Black: 31.37 ± 0.69, Non-black: 28.29 ± 0.23, P < 0.004, Kruskal-Wallis with Dunn’s post-test) with type 1 (Black: 31.58 ± 0.86, Non-black: 27.82 ± 0.29, P < 0.001, Kruskal-Wallis with Dunn’s post-test) diabetes. We did not have enough black/African American individuals in the type 2 diabetic gastroparesis group to power the comparison in that group. After correcting for this disparity in the distributions of the allele lengths among racial groups, no significant differences were detected between the allele lengths in non-black individuals with and without gastroparesis.

### Association of HMOX1 polyGT allele lengths and gastroparesis symptoms

**Fig 4:**
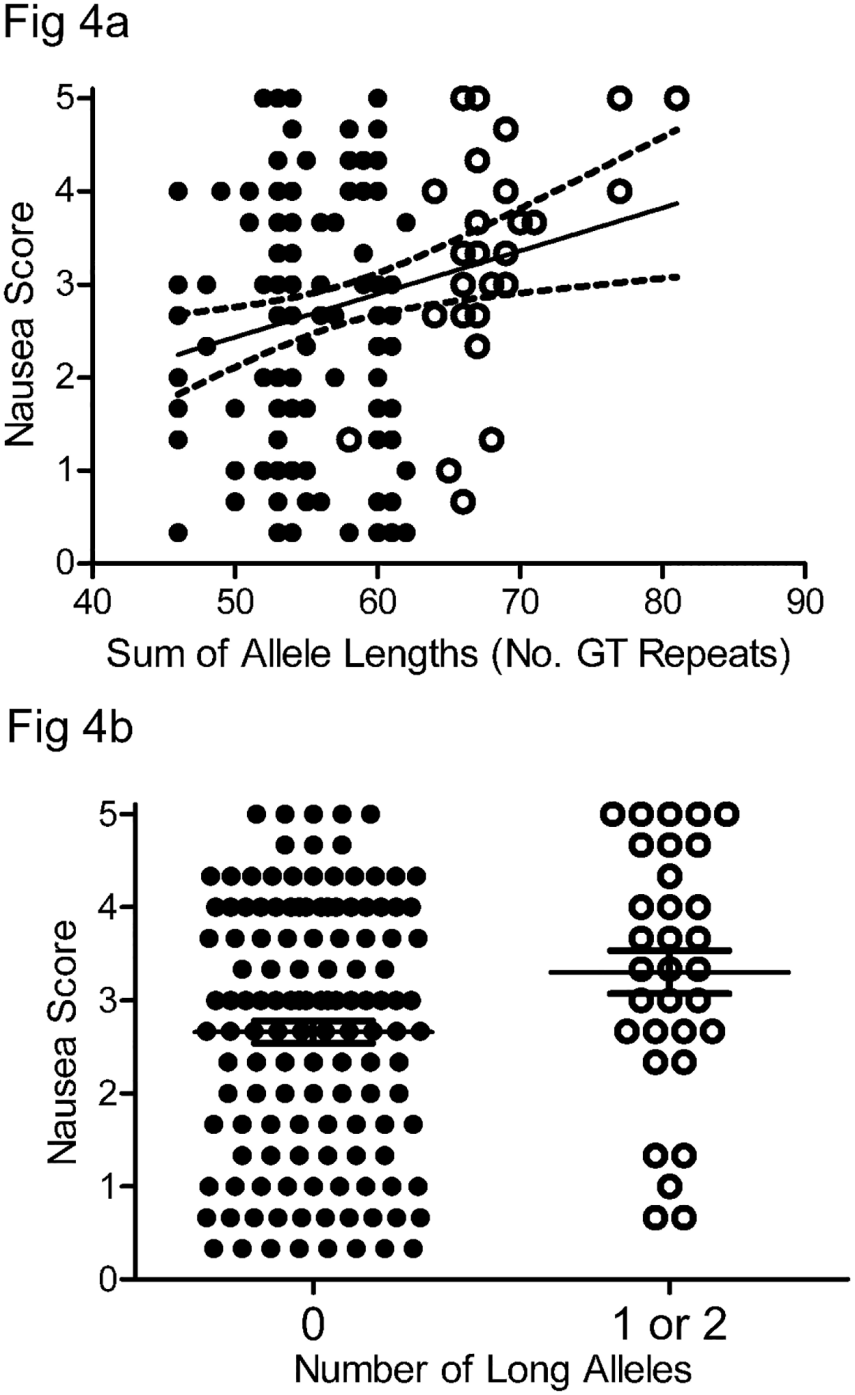
Relationship between polyGT allele length and nausea sub-score. In patients with diabetic gastroparesis, the relationship between polyGT allele length and nausea sub-score on the GCSI questionnaire fit to a slope that was significantly different from zero (P < 0.006 by linear regression, dotted lines are 95% confidence intervals) and b) subjects with one or two long alleles (open circles in Fig 4a) had significantly higher nausea sub-scores than subjects with zero long alleles (closed circles in Fig 4a, P = 0.022, Mann Whitney test). Whiskers are the medians with the interquartile ranges for the nausea sub-score.

To investigate possible associations between the length of the polyGT repeat allele in the *HMOX1* gene with symptoms of gastroparesis, we plotted either the length of the shorter of the two alleles, the length of the longer of the two alleles or sum of the two allele lengths in each subject against each of the measured variables and then fitted the data by linear regression. We found no relationships between allele length and BMI, HbA1c levels, or gastric emptying for either diabetic or idiopathic gastroparesis. Additionally, the overall GCSI and sub-scores for fullness and bloating were not associated with allele lengths. However, there was a significant association between longer total allele length and GCSI nausea sub-score in patients with diabetic gastroparesis (P < 0.006, Fig 4a). A significant association was also evident when plotting the length of the longer allele against the nausea sub-score but not when plotting the length of the shorter allele against the nausea sub-score. The majority of diabetic gastroparesis subjects with one or two long alleles (open circles Fig 4a) had nausea sub-scores ≥ 3, which is considered severe and the average nausea sub-scores were significantly higher in diabetic gastroparetic subjects with one or more long alleles compared to subjects with no long alleles (Fig 4b, P = 0.022, Mann Whitney test). There was no association between allele length and nausea symptoms in idiopathic gastroparetic patients.

Since African American individuals with gastroparesis tended to have longer polyGT alleles, we hypothesized that African American individuals with gastroparesis might be predicted to have worse nausea sub-scores than non-African Americans with gastroparesis. This proved to be correct, the nausea sub-scores in Black/African American individuals with gastroparesis were significantly higher that the scores for non-black individuals with gastroparesis (Black: 3.33, 1.83–4, Non-black: 2.5 1.33–3.67, Mean, IQR, P = 0.05, Mann Whitney test).

## Discussion

The principal finding of this study is the presence of longer poly GT repeat alleles in the *HMOX1* genes of black/African American subjects with gastroparesis compared to the alleles in non-black/African American subjects with gastroparesis. This difference accounts for differences in allele lengths between subjects with gastroparesis and non-diabetic, non-gastroparetic subjects. Furthermore, we found that in subjects with diabetic gastroparesis, nausea symptoms were significantly worse in subjects with one or two long polyGT repeat alleles when compared to subjects with zero long polyGT repeat alleles and that in black/African American individuals with gastroparesis, nausea sub-scores were worse than the scores in non-blacks with gastroparesis. These findings raise the important issue of ensuring that race/ethnicity is taken into account for these kinds of studies and stand in contrast with studies on a variety of diseases that showed worse outcomes and more severe symptoms in people with longer polyGT repeat alleles in their *HMOX1* genes including type 2 diabetes [15], emphysema [16], Parkinson’s Disease [17], ischemic heart disease [18] and coronary artery disease in subjects with type 2 diabetes [19].

Previous studies have reported that up to 30% of alleles in African American and West African populations contain very long repeats (peaking at around 39 repeats), whereas in the non-black individuals in our study and in other populations from Brazil, Japan, Thailand and Greece less than 3% of alleles are as long as 39 repeats [37, 38]. Also African American and West African populations seem to have 6% or fewer short polyGT repeats that are 23 repeats long compared to other populations where 20% or more alleles are <23 repeats long [37]. A small number of African American individuals in our study with very long repeats significantly skewed the data to suggest an association of longer polyGT repeats with gastroparesis in the population as a whole. The difference in average allele length of approximately 3 repeats between black/African American individuals and non-blacks in all the groups that we studied correlates with a change in HMOX1 promoter activity and HO1 protein activity of as much as 50% based on at least one published study [24]. This observation suggests that polymorphisms in the HMOX1 gene could be one contributing factor to racial disparities in diseases such as coronary artery disease and Type 2 diabetes [39, 40], which have been associated with differences in the length of the polyGT repeat in this gene [15, 19]. Large, fully powered studies that take into account race are necessary to compare the allelic differences in the HMOX1 gene in all of these diseases including gastroparesis, where there is a rationale to consider a role for HO1 in the prevalence and progression of the disease.

The choice of cut-offs for this study between short, medium and long allele lengths was made based on the distribution in the polyGT allele lengths for the promoter region of *HMOX1* gene in our data from the control subjects. We chose a 3 allele length model to test the distribution of the allele lengths since this was the most common approach in other studies and was consistent with the distribution of our data. There is no consensus in the literature for these cutoff values (for a discussion see [15]). Some groups have chosen a two factor, short versus long model [17, 41], which simplifies analysis but requires a choice as to whether to include the majority of the allele lengths in the 29–32 range with the short alleles or with the long alleles. Other groups used the same three component model as we did, with alleles being consistently considered long if containing greater than 32 polyGT repeats [16, 33, 35] and with a variety of choices made with respect to the boundary between short and medium. As is evident from Fig 1a, a 4 component model might more accurately reflect the data, with alleles in the range of 25–29 repeats placed in a separate group, but that would come at a significant cost in terms of reduced power to analyze the data and much greater complexity in the analysis. This 25–29 range has been defined a medium length allele in a 3 component model that was based on HO1 protein activity [24], and does correspond to a polyGT repeat allele length that is prominent in the HMOX1 genes of African Americans with HIV [36] and West Africans [37, 38] but not in the populations that we studied. Therefore we used a widely accepted 3 component model and assigned the alleles < 29 polyGT repeats to the short category. The robustness of the observations are supported by the data confirming previous studies [15] in showing a significantly higher abundance of long polyGT alleles in *HMOX1* of subjects with type 2 but not type 1 diabetes, when compared to non-diabetic controls. We did not detect a difference, if present, between subjects with type 2 diabetes and gastroparesis and subjects with type 2 diabetes and no gastroparesis and we did not find a significant correlation between BMI in the non-diabetic subjects and polyGT allele length in *HMOX1*.

Gastroparesis is a syndrome defined by an associated group of symptoms that present to differing degrees in affected patients as analyzed in detail by the Gastroparesis Clinical Research Consortium [32] for a subset of the patients examined here for variations in the polyGT allele of *HMOX1*. However, there is no clear relationship between a particular symptom profile and any underlying cause of gastroparesis. The association of worse nausea symptoms with the presence of longer polyGT repeat alleles in *HMOX1* of patients with diabetic but not idiopathic gastroparesis is a finding of interest because nausea and vomiting are symptoms that are reported to be more common in patients evaluated for diabetic gastroparesis whereas abdominal pain is a more common symptom in patients evaluated for idiopathic gastroparesis [2, 42]. For this study, the patients with idiopathic gastroparesis did have lower nausea sub-scores than either diabetic group but this difference was not significant and did not impact our ability to detect an association between the nausea sub-score and polyGT allele length in *HMOX1*. Nausea and vomiting are also symptoms of other functional gastrointestinal disorders that do not involve delayed gastric emptying including chronic un-explained nausea and vomiting (CUNV) [43]. The underlying cytopathology in CUNV appears similar to that found in gastroparesis including loss of ICC [44]) therefore if a sufficiently large cohort of patients with CUNV can be obtained; it will be informative to investigate whether polyGT allele length in the *HMOX1* promoter associates with symptom severity in CUNV. We carefully excluded this population from our control groups. The association of worse nausea symptoms with the length of the longer allele as opposed to the shortness of the shorter allele appears to indicate that long total allele lengths and very low HO1 levels pre-dispose to nausea symptoms, as opposed to the possibility that shorter short alleles provide incrementally more protection from symptoms. This is in contrast to a study on susceptibility to severe malaria in children, where the shortness of the short alleles rather than length of the long alleles was associated with worse outcomes [38] linking high HO1 levels to greater susceptibility to hemolytic diseases.

There is an established role for HO1 in protecting or restoring gastric muscularis propria function from diabetic injury in animal models. In diabetic mice with delayed gastric emptying, HO1 is up-regulated following onset of diabetes but absent in a subset of mice that developed delayed gastric emptying [14]. Induction of HO1 expression by treatment with hemin [14] or IL10 [25] of diabetic mice with delayed gastric emptying restored gastric emptying to normal and resulted in normalization of markers for ICC (Kit) and inhibitory neurotransmission (nNOS). In mice, HO1 is expressed in CD206^+^ alternatively activated macrophages [45], a subset of anti-inflammatory macrophages that are depleted in patients with diabetic gastroparesis, delayed gastric emptying and profound loss of ICC [46]. Therefore we proposed that diverse promoter sequences in the *HMOX1* gene might contribute to differences between individuals in the development of gastroparesis in the context of diabetes by changing the amount HO1 protein generated in alternatively activated macrophages in response to tissue damage, oxidative stress or other cytotoxic factors. In this respect, gastroparesis is similar to coronary artery disease, a complication of diabetes that has also been shown, in a study on a Chinese population with type 2 diabetes, to be more prevalent in people who have long GT repeats in their *HMOX1* genes [19]. However, it appears that although the specific symptoms of nausea and vomiting are more prominent in individuals with gastroparesis who have long alleles, our study indicates that black/African American individuals with gastroparesis have longer polyGT alleles and have worse nausea symptoms than non-black individuals with gastroparesis, although we did not have the power to determine if the nausea symptoms correlate with polyGT allele in a significant fashion. It is likely that several hits lead to development of gastroparesis and long poly(GT) repeats in *HMOX1* may represent one of these hits especially when the allele length is so long that it leads to very low basal and inducible HO1 protein activity [24].

The evidence that both carbon monoxide and biliverdin are effective cytoprotective molecules in animal models of gastrointestinal injury [47, 48] and other diseases is extensive [49, 50]. In contrast, the mechanism by which the long polyGT repeat allele in the promoter for *HMOX1* can contribute to a greater susceptibility to disease and injury has not been as fully established. In heterologous expression studies on luciferase expression in cell lines, it appears that the transcriptional activity of the *HMOX1* promoter is lower when the promoter contains more GT repeats with respect to transcriptional activity at baseline[19, 24] and in response to oxidative stress induced by hydrogen peroxide [16]. Thus, the long polyGT allele in the *HMOX1* promoter can reduce HO1 transcription and can interfere with capacity of tissues to respond to oxidative injury [20]. In contrast, short polyGT repeat alleles and high HO1 activity levels have been associated with worse outcomes following malaria infection in some [38] but not all [37] studies on West African populations. This has been proposed to be a selective pressure for evolutionary selection of longer polyGT repeat alleles in the HMOX1 genes of ethnic groups exposed to hemolytic illnesses such as individuals in West Africa [51]. The long polyGT allele could also impact the response of patients to therapies targeted at exploiting the cytoprotective effect of up-regulated HO1 activity. Further research on the mechanistic effect of the polyGT repeat is required, particularly with the identification of further complexity in the transcriptional regulation of *HMOX1* expression, which is influenced by both the polyGT repeat and an adjacent single nucleotide polymorphism upstream of the polyGT repeat [52]. Therefore there are both potential mechanisms and experimental evidence that can explain how a longer polyGT allele in *HMOX1* can pre-dispose an individual to the cellular changes that lead to gastroparesis in the presence of high glucose, advanced glycation end products, oxidative stress and other markers of diabetes. Further investigations into the mechanisms by which the long polyGT repeat allele suppresses HO1 activity are necessary with a focus on understanding the transcriptional regulation of *HMOX1*, a gene that encodes a protein with powerful and important biological effects.

Our data do not support a strong link between the poly GT repeat allele in *HMOX1* and idiopathic gastroparesis despite the similarities in the pathological changes observed in diabetic and idiopathic gastroparesis of humans [29]. This may reflect diverse causes of idiopathic gastroparesis or a lack of power due to the diversity of the large group of idiopathic gastroparetic subjects. This study indicates that HO1 activity is a factor that might contribute to a subset of symptoms in a subset of patients specifically individuals of African origin with diabetic gastroparesis.

There were a number of significant differences in the demographics of our control and gastroparetic groups. The subjects in the control groups were older than the gastroparetic groups and the non-gastroparetic, non-diabetic control group included a higher proportion of male subjects. We selected older control subjects with no history of symptoms of gastrointestinal motility disorders including no history of gastroparesis or nausea and vomiting. The rationale for this was to allow sufficient time for the impact of polyGT allele length on symptoms of gastroparesis to become evident. There is no evidence that healthy aging overall depends on this allele so we do not think that the age difference had a significant impact on our observations although there might be a small influence if there were an, as yet unproven, higher probability of survival in individuals with shorter alleles. Although there is evidence of gender bias in the distribution of this allelic variant in *HMOX1* with respect to development of lung adenocarcinoma in Japanese smokers [34], there was no difference in the allele lengths between males and females in any of the groups from our study, so we do not think that the differences in the proportions of males and females between the groups had an impact on our observations.

## Conclusions

Overall, the implications of this study are that longer poly-GT repeats in the *HMOX1* gene are more common in African Americans with gastroparesis and represent a potential risk factor for gastroparesis in this group since very long repeat alleles predispose those people to both gastroparesis as well as coronary artery disease as reported in a previous study [19]. As the power of genome sequencing increases and becomes more widely available, the knowledge of susceptibility alleles will increase, raising the possibility of targeting people with long alleles and who exhibit metabolic syndrome or are in early stages of diabetes to receive more aggressive treatment with a focus on the symptoms of nausea or vomiting to prevent development of disabling complications.

## Supporting information

S1 File A file containing the minimal data necessary to replicate these studies. (XLSX)
